# A Proof-of-Concept Free-Flight Photogrammetric Framework Based on Monocular Vision and Sensor-Group Displacement Fusion

**DOI:** 10.3390/s26103177

**Published:** 2026-05-17

**Authors:** Enshun Lu, Xin Wan, Wupeng Deng, Xiaofeng Li

**Affiliations:** 1 School of Agricultural Machinery Engineering Research and Design Institute, Hubei University of Technology, Wuhan 430068, China; gundamresearcher@163.com (E.L.); wanxinlunar@163.com (X.W.); jellyli0506@163.com (X.L.); 2 School of Mechanical Engineering, Hubei University of Technology, Wuhan 430068, China

**Keywords:** free-flight photogrammetry, sensor group, information fusion, rigid point set resection, composite weighting

## Abstract

As unmanned aerial vehicles (UAVs) have increasingly become aerial imaging platforms, the reliance of traditional photogrammetry on ground control points (GCPs) remains a major limitation in complex terrain, confined spaces, and scenarios where control points are difficult to deploy. To address this issue, this study proposes a proof-of-concept framework for free-flight photogrammetry based on the fusion of monocular vision and sensor-group displacement information. The framework employs a rigid point set station-displacement algorithm to compute the exterior orientation elements between adjacent measurement stations, providing a feasible approach for multi-station pose propagation under control-point-free conditions. In addition, a composite weighting strategy incorporating the effects of optical distortion and rigid-body consistency evaluation is developed to improve the rational use of point-set information during station-displacement computation. To evaluate the feasibility of the proposed method, numerical simulations were first conducted to analyze the variation patterns of exterior orientation computation and target-point reconstruction under different sampling intervals and error conditions. Subsequently, an indoor controlled bench-top experimental platform was constructed to physically validate the complete workflow of the proposed method. The bench-top experimental results show that the overall mean three-dimensional positioning error of the two cross-station image pairs was 15.450 mm, and the maximum three-dimensional positioning error was 36.685 mm. The mean absolute distance errors for station 1–station 2 and station 1–station 3 were 9.230 mm and 12.436 mm, respectively. These results indicate that the proposed method can complete station-displacement-based exterior orientation computation and three-dimensional target measurement in a controlled physical scenario, demonstrating clear proof-of-concept significance. It should be noted that UAV measurement experiments under real flight conditions have not yet been completed in this study, and further validation on an actual UAV platform is still required.

## 1. Introduction

As a non-contact measurement technique that excels in measurement accuracy, automation, and cost control, photogrammetry has attracted increasing attention in recent years from industries such as ports [1,2,3], urban viaduct construction [4,5], shipbuilding [6], and defense [7].

In these industries, the predominant method is close-range photogrammetry using fixed measurement stations. While this method can meet certain engineering measurement requirements, its application is inherently limited in scenarios that lack stable platforms—such as densely packed port environments, viaduct construction sites located in remote mountainous areas, or shipyards without elevated footholds [8,9].

In recent years, the advancement of flight control technology has effectively addressed the limitations of close-range photogrammetry in covering complex engineering scenarios. However, traditional aerial photogrammetry imposes stringent requirements on image overlap—typically requiring no less than 60% forward overlap and 20% lateral overlap—thereby placing significant constraints on the flight paths of aerial platforms [10]. In engineering sites such as ports, the significant variation in object heights and the highly dynamic stacking environments make it difficult to provide a safe and sufficiently close clearance zone for aerial photography. As a result, continued reliance on traditional fixed-flight-path photography limits the proximity of measurement stations to target objects, thereby compromising measurement accuracy.

At present, UAV-based vision measurement systems typically use rigid frames to mount multiple cameras, with key parameters such as camera intrinsic matrices and exterior orientation elements being pre-calibrated in the lab. In effect, this transforms traditional aerial photogrammetry into a mobile POS-based close-range photogrammetry system.

Due to the limitations in size and payload capacity of unmanned aerial vehicles (UAVs), it is difficult to ensure adequate baseline length for POS (Position and Orientation System) systems. As a result, current UAV-based free-flight photogrammetry methods face the following issues: (1) the lack of a suitable imaging baseline for long-distance measurements leads to generally unsatisfactory measurement accuracy; (2) these systems are also unsuitable for high-altitude free-flight missions, again due to the insufficient baseline length. To address these challenges, this study explores a UAV-compatible free-flight photogrammetry approach aimed at reconciling the trade-off between measurement accuracy and equipment portability.

The remainder of this paper is organized as follows: Section 2 introduces the exterior orientation parameters solving method based on rigid point set station displacement, along with a composite weighting-based algorithm for optimizing displacement accuracy. Section 3 presents numerical simulations to examine the mathematical consistency of proposed method, while Section 4 provides a controlled indoor bench-scale proof-of-concept experiment. Finally, the conclusions are presented in Section 5.

## 2. Methodology

According to the classical photogrammetric process, to achieve the conversion from the planar coordinates of points on photos to the corresponding three-dimensional coordinates in the object coordinate system, the main computational steps include: (1) identification of the points to be measured and completion of the matching of homologous points; (2) computation of the exterior orientation elements of each measurement station through resection; (3) computation of the exterior orientation elements through forward intersection, based on the completed computation of the exterior orientation elements, to accomplish the conversion of coplanar two-dimensional coordinates to three-dimensional coordinates in the object coordinate system.

However, in real-world environments, the number of control points may be insufficient or even completely unavailable. Moreover, identifying control points and matching corresponding points in the image plane coordinate system demands substantial computational power, inevitably causing a significant negative impact on the system’s overall execution efficiency. Therefore, traditional photogrammetric methods for solving exterior orientation elements that rely on control points are not suitable for UAV vision measurement applications.

This paper introduces an independently developed technical alternative: an exterior orientation element calculation method based on rigid point set resection.

### 2.1. The Method for Computing Exterior Orientation Elements Based on the Displacement of a Rigid Point Set

After installing several accelerometers inside the UAV, one of the two adjacent measurement stations can be used as a reference to directly calculate the relative displacement of the rigid point set formed by the sensor centers between the two measurement stations.

Since the UAV can rely on the navigation system for positioning to achieve synchronization with the geodetic coordinate system or to reset the coordinates of the initial measurement station to zero during flight, it can be assumed that the object-space 3D coordinates of each rigid point in the rigid point set are known at any moment during the UAV’s flight.

Definition 2-1: Rigid Point Set Assumption

Although displacement inevitably exists between the points formed by sensors due to sensor errors and other factors, we still assume during the calculation process that they remain a perfect rigid body at each measurement station, referring to them as a rigid point set.

**Figure 1:**
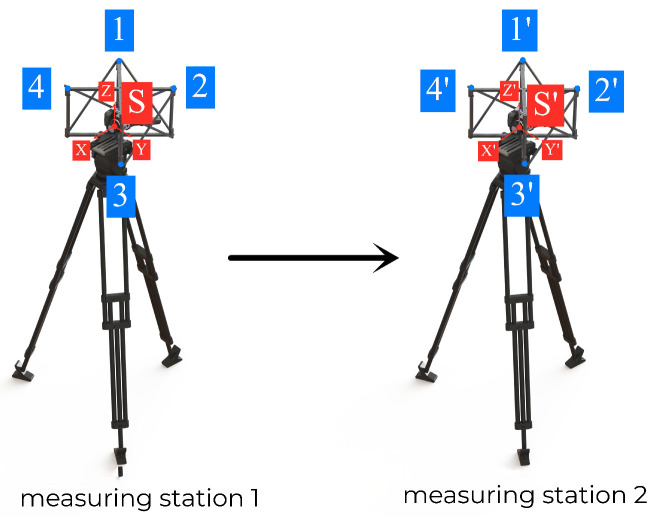
Schematic diagram of station displacement principle based on rigid point set.

On this basis, the calculation of the measurement station parameters can be completed. As shown in Figure 1, the object-space coordinates determined based on point 1, point 2, point 3 and point 4 at measurement station 1, and point $1^{\prime }$, point $2^{\prime }$, point $3^{\prime }$ and point $4^{\prime }$ at measurement station 2 That is, the rotation vector $R_{s}$ and translation vector $S_{s}$ between the coordinate systems S−XYZ and $S^{\prime }-X^{\prime }Y^{\prime }Z^{\prime }$ that are connected to it can be calculated.

**Figure 2:**
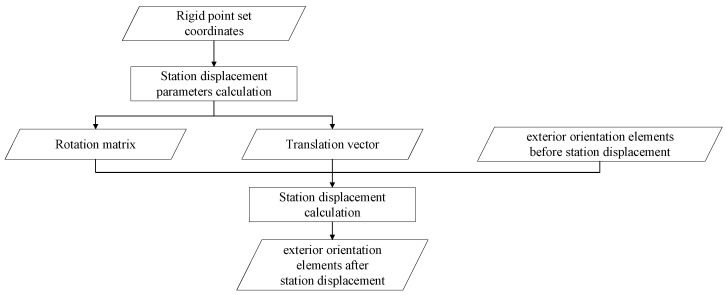
Schematic diagram of the station displacement calculation process.

Since the relative positional relationship between the photographic center of the measurement station and the rigid point set is fixed. Therefore, the rotation vector $R_{s}$ and the translation vector $S_{s}$ between S−XYZ and $S^{\prime }-X^{\prime }Y^{\prime }Z^{\prime }$ are similarly applicable for the displacement calculation of exterior orientation elements between measurement station 1 and measurement station 2. Using the process shown in Figure 2, the exterior orientation elements calculation without control points can be realized, thus greatly improving the adaptability of the monocular photogrammetry algorithm.

The following is the mathematical implementation process of the station displacement algorithm. The detailed MATLAB implementation code for reproducing the station-displacement calculation is provided in Supplementary Materials.

#### 2.1.1. Solving Conditions

The known conditions for solving the exterior orientation elements of measurement station 2 include:

(1) The exterior orientation elements of measurement station 1 are $\left[X_{1},Y_{1},Z_{1},\varphi_{1},\omega_{1},\kappa_{1}\right]$.

(2) The rigid point set contains a total of n (n≥3) points. The object-space coordinates of each point at measurement station 1 are: $\left(X1_{i},Y1_{i},Z1_{i}\right)$,i=1,2,…,n, their values can be measured by the mounting platform or specified manually.

(3) The object-space coordinates of each point at measurement station 2 are: $\left(X2_{i},Y2_{i},Z2_{i}\right)$, i=1,2,…,n, Their values are synthesized from the displacement measured by the acceleration sensors and the initial values at measurement station 1.

The solution target is the exterior orientation elements of measurement station 2: $\left[X_{2},Y_{2},Z_{2},\varphi_{2},\omega_{2},\kappa_{2}\right]$.

#### 2.1.2. Exterior Element Line Elements Solution

Let P be the set of object-space coordinates of the rigid point set of measurement station 1, and Q be the set of object-space coordinates of the rigid point set of measurement station 2:

(1) $$P=\left[\begin{array}{cccc} X1_{1} & X1_{2} & \ldots & X1_{n}\\ Y1_{1} & Y1_{2} & \ldots & Y1_{n}\\ Z1_{1} & Z1_{2} & \ldots & Z1_{n} \end{array}\right].$$

(2) $$Q=\left[\begin{array}{cccc} X2_{1} & X2_{2} & \ldots & X2_{n}\\ Y2_{1} & Y2_{2} & \ldots & Y2_{n}\\ Z2_{1} & Z2_{2} & \ldots & Z2_{n} \end{array}\right]\;.$$

Then the rotation vector $R_{s}$ and translation vector $S_{s}$ that can realize the conversion of matrix P to matrix Q should meet the following conditions:

(3) $$\left\{\begin{array}{c} Q=R_{S}\cdot P+S_{S}\\ F\left(R_{S},S_{S}\right)=\arg\min\sum_{i=1}^{n}{\left|\left(R_{S}P_{i}+S_{S}\right)-Q_{i}\right|}^{2} \end{array}\right.$$

From Formula (3) we can know:

(4) $$\begin{array}{c} \frac{\partial F}{\partial S_{S}}=2\sum_{i=1}^{n}\left[\left(R_{S}P_{i}+S_{S}\right)-Q_{i}\right]\\ =2nS_{S}+2R_{S}\sum_{i=1}^{n}P_{i}-2\sum_{i=1}^{n}Q_{i}\;. \end{array}$$

Let the center point of set P is $P_{Z}\left(X_{PZ},Y_{PZ},Z_{PZ}\right)$, and the center point of set Q is $Q_{Z}\left(X_{QZ},Y_{QZ},Z_{QZ}\right)$, they meet the conditions:

(5) $$\left\{\begin{array}{cc} {}[X_{PZ},Y_{PZ},Z_{PZ}]\; & =\left[\frac{1}{n}\sum_{i=1}^{n}X_{1i},\;\frac{1}{n}\sum_{i=1}^{n}Y_{1i},\;\frac{1}{n}\sum_{i=1}^{n}Z_{1i}\right],\\ {}[X_{QZ},Y_{QZ},Z_{QZ}]\; & =\left[\frac{1}{n}\sum_{i=1}^{n}X_{2i},\;\frac{1}{n}\sum_{i=1}^{n}Y_{2i},\;\frac{1}{n}\sum_{i=1}^{n}Z_{2i}\right]. \end{array}\right.$$

Obviously, the center point also satisfies the conditions:

(6) $$Q_{Z}=R_{S}\cdot P_{Z}+S_{S}\Rightarrow S_{S}=Q_{Z}-R_{S}\cdot P_{Z}\;.$$

By combining Formula (3) and Formula (6), we can get:

(7) $$\sum_{i=1}^{n}{\left|\left(R_{S}P_{i}+S_{S}\right)-Q_{i}\right|}^{2}=\sum_{i=1}^{n}{\left|R_{S}\left(P_{i}-\bar{P}\right)-\left(Q_{i}-\bar{Q}\right)\right|}^{2}\;.$$

(8) $$\begin{array}{c} F\left(R_{S},S_{S}\right)=\arg\min\sum_{i=1}^{n}{\left|R_{S}x_{i}-y_{i}\right|}^{2}\\ =x_{i}^{T}x_{i}-y_{i}^{T}R_{S}x_{i}-x_{i}^{T}R_{S}^{T}y_{i}+y_{i}^{T}y_{i}\;. \end{array}$$

Among them: $x_{i}=P_{i}-\bar{P}$, $y_{i}=Q_{i}-\bar{Q}$.

In addition, based on the scalar properties, it can be deduced: $x_{i}^{T}R_{S}y_{i}={\left(x_{i}^{T}R_{S}y_{i}\right)}^{T}=y_{i}^{T}R_{S}x_{i}$, Formula (8) can also be simplified to:

(9) $$\begin{array}{cc} \; & F\left(R_{S},S_{S}\right)=\arg\min\sum_{i=1}^{n}{\left|R_{S}x_{i}-y_{i}\right|}^{2}\\ \; & =\arg\min\sum_{i=1}^{n}\left(x_{i}^{T}x_{i}-2y_{i}^{T}R_{S}x_{i}+y_{i}^{T}y_{i}\right)\;. \end{array}$$

On this basis, since $x_{i}^{T}x_{i}$ and $y_{i}^{T}y_{i}$ and $R_{S}$, $S_{S}$ is irrelevant, the following inference can also be drawn:

(10) $$\begin{array}{cc} \; & F\left(R_{S},S_{S}\right)=\arg\min\left(-2\sum_{i=1}^{n}y_{i}^{T}R_{S}x_{i}\right)\\ \; & =\arg\max\left(\sum_{i=1}^{n}y_{i}^{T}R_{S}x_{i}\right)\;. \end{array}$$

(11) $$\begin{array}{cc} \; & Y^{T}R_{S}X=\left[\begin{array}{c} {y_{1}}^{T}\\ {y_{2}}^{T}\\ \vdots \\ y_{n}^{T} \end{array}\right]R_{S}\left[\begin{array}{cccc} x_{1} & x_{2} & \ldots & x_{n} \end{array}\right]\\ \; & =\left(\begin{array}{ccc} y_{1}^{T}R_{S}x_{1} & \; & \\ \; & {y_{2}}^{T}R_{S}x_{2} & \\ \; & \; & \\ \; & \ldots & \\ \; & \; & {y_{n}}^{T}R_{S}x_{n} \end{array}\right)\;. \end{array}$$

Among them: $X=[x_{1},x_{2},\ldots ,x_{n}]$, $Y=[y_{1},y_{2},\ldots ,y_{n}]$.

Based on Formulas (10) and (11):

(12) $$\begin{array}{cc} \; & \sum_{i=1}^{n}y_{i}^{T}R_{S}x_{i}=\mathrm{tr}\left(Y^{T}R_{S}X\right)=\mathrm{tr}\left(\left(Y^{T}\right)\left(R_{S}X\right)\right)\\ \; & =\mathrm{tr}\left(\left(R_{S}X\right)\left(Y^{T}\right)\right)=\mathrm{tr}\left(R_{S}XY^{T}\right)\;. \end{array}$$

Let $S=XY^{T}$, perform singular value decomposition on the matrix S to get:

(13) $$S=U\Sigma V^{T}\;.$$

Among them, matrices U and V are orthogonal matrices, and matrix Σ is a diagonal matrix.

By combining Formulas (12) and (13), we can get:

(14) $$\begin{array}{cc} \; & \mathrm{tr}\left(R_{S}XY^{T}\right)=\mathrm{tr}\left(R_{S}S\right)=\mathrm{tr}\left(R_{S}U\Sigma V^{T}\right)\\ \; & =\mathrm{tr}\left(\Sigma V^{T}\right)\left(R_{S}U\right)=\mathrm{tr}\left(\Sigma V^{T}R_{S}U\right)\;. \end{array}$$

Let $M=V^{T}R_{S}U$, obviously the column vector $m_{j}$ contained in the matrix M satisfies the condition: ${m_{j}}^{T}m_{j}=1$, by This can be deduced:

(15) $$\begin{array}{cc} \mathrm{tr}(\Sigma M) & =\mathrm{tr}\;\left(\mathrm{diag}(\sigma_{1},\sigma_{2},\ldots ,\sigma_{d})\;M\right)\;=\sum_{i=1}^{d}\sigma_{i}m_{ii}\leq \sum_{i=1}^{d}\sigma_{i}\;m_{i}^{T}m_{i}\;=\sum_{i=1}^{d}\sigma_{i}. \end{array}$$

When $M=I=V^{⊤}RU$, you can get $\mathrm{Max}\left(\sum_{i=1}^{n}y_{i}^{T}R_{S}x_{i}\right)$.

At this point, we can complete the solution of the rotation vector $R_{S}$ and the translation vector $S_{S}$:

(16) $$R_{S}=VU^{T}\;.$$

(17) $$S_{S}=\bar{Q}-R_{S}\bar{P}\;.$$

It can be seen that the line element conversion relationship between measurement station 1 and measurement station 2 is:

(18) $$\left(\begin{array}{c} X_{2}\\ Y_{2}\\ Z_{2} \end{array}\right)=R_{S}\cdot \left(\begin{array}{c} X_{1}\\ Y_{1}\\ Z_{1} \end{array}\right)+S_{S}\;.$$

According to Formula (18) and the calculation method of angular elements in the classic photogrammetry algorithm, the exterior orientation elements: $\left[\begin{array}{c} X_{2},Y_{2},Z_{2},\varphi_{2},\omega_{2},\kappa_{2} \end{array}\right]$ of measurement station 2 can be completed, enabling the solution of exterior orientation elements based on the rigid point set resection that does not rely on control points. This method provides a theoretical foundation for UAV monocular vision measurement based on sensor data.

### 2.2. The Precision Optimization Algorithm Based on Composite Weighting for Station Displacement

The foundation established for Formula (15) assumes that all points in the rigid point set have equal weights during the process of station displacement. However, based on the principle of uncertainty, in actual measurement processes, each rigid point inevitably contains different measurement errors. Assigning them equal weights during the process of solving exterior orientation elements through station displacement does not meet the rigorous scientific requirements.

Therefore, it is necessary, as shown in Formula (19), to introduce a weighting coefficient matrix W during the solving process. By quantitatively assessing the errors contained in each point participating in the calculation of the rigid point set, scientific weighting is applied. This approach is intended to provide a more reasonable use of redundant point observations and to improve the stability of the station-displacement estimation under the assumed error model.

(19) $$Y^{T}R_{S}X=W\cdot \left[\begin{array}{c} y_{1}^{T}\\ y_{2}^{T}\\ \vdots \\ y_{n}^{T} \end{array}\right]R_{S}\left[\begin{array}{cccc} x_{1} & x_{2} & \ldots & x_{n} \end{array}\right]$$

(20) $$W=\left[\begin{array}{cccc} Q_{1} & \; & & \\ \; & Q_{2} & \; & \\ \; & \; & \ldots & \\ \; & \; & & Q_{n} \end{array}\right]\;.$$

Among them, $Q_{i}$ is the weight of each point of the rigid point set in the solution model, i=1,2,…,n, n is the number of points included in the rigid point set, n≥3.

The factors influencing the error level of each point mainly include:

(1) Errors caused by optical distortion of the camera lens:

Due to inevitable errors in the manufacturing and assembly process, image point offsets caused by optical distortion cannot be avoided at this stage. Furthermore, this type of error changes with the position of the image points in the image plane coordinate system, directly affecting the weights of each point in the solution model.

(2) Decrease in the consistency of the rigid point set due to sensor errors:

In the UAV’s actual vision measurement process, the formation of the rigid point set mainly relies on data collected from accelerometers. However, due to random measurement errors from the sensors, the reconstructed object-space point set at each measurement station cannot form a strictly rigid structure. Additionally, deformation and vibrations of the UAV body, along with possible random displacements of system components, further undermine the assumption of a perfect rigid body. Since the station-displacement calculation process heavily depends on the rigidity assumption of the point system, it is crucial to reasonably assign weights to each point in the calculation model based on how data errors affect the system’s rigidity. This weighting strategy is expected to reduce the influence of low-consistency points on the station-displacement solution, although its effectiveness still requires further validation under real flight conditions.

#### 2.2.1. Weighting Algorithm Based on Optical Distortion

In traditional photogrammetry, precise calibration can be used to obtain relevant parameters and compensate for corresponding errors through correction algorithms. However, during UAV flight, external disturbances such as wind and airflow can cause the UAV to experience turbulence, making it difficult to ensure the timeliness of laboratory calibration results. Additionally, the calibration process often relies on specialized calibration facilities, which can be costly.

For these reasons, the technical solution adopted in this paper does not rely on distortion parameter correction algorithms. Instead, it applies weighting to the calculation model based on recommended calibration distortion parameters, thereby improving the accuracy of exterior orientation elements station-displacement calculations within a reasonable budget.

**Figure 3:**
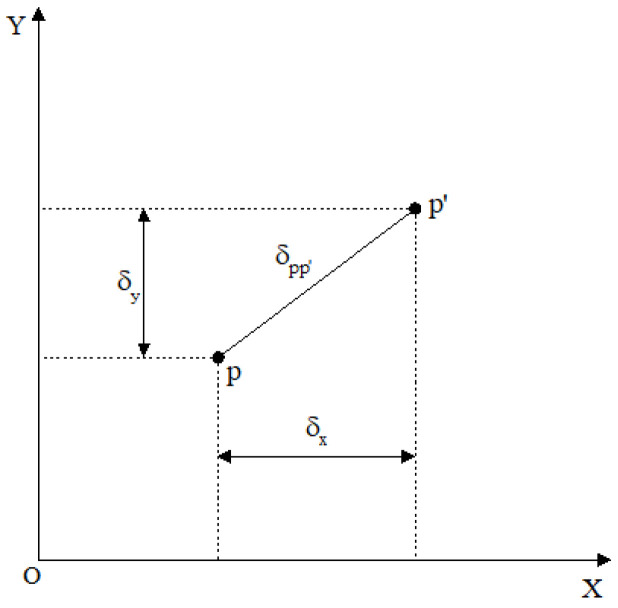
Schematic diagram of optical distortion synthesis form.

As shown in Figure 3, various distortions are displayed in the form of the displacement of the image point on each photo within the image plane coordinate system. In the image plane coordinate system, the displacement deviation model in the x-axis and y-axis directions caused by optical distortion is [11,12,13,14]:

(21) $$\begin{array}{cc} \; & \delta_{x}=\delta_{jx}+\delta_{lx}+\delta_{bx}\\ \; & =k_{1}x\left(x^{2}+y^{2}\right)+\left[q_{1}\left(3x^{2}+y^{2}\right)+2q_{2}xy\right]+s_{1}\left(x^{2}+y^{2}\right)\;. \end{array}$$

(22) $$\begin{array}{cc} \; & \delta_{y}=\delta_{jy}+\delta_{ly}+\delta_{by}\\ \; & =k_{2}y\left(x^{2}+y^{2}\right)+\left[q_{2}\left(3x^{2}+y^{2}\right)+2q_{1}xy\right]+s_{2}\left(x^{2}+y^{2}\right)\;. \end{array}$$

Among them: $\delta_{x}$, $\delta_{y}$ are the values of the nonlinear distortion synthesis results in the X-axis and Y-axis directions of the image plane coordinate system respectively; $\delta_{jx}$, $\delta_{jy}$ are respectively the radial distortion parameters in the X-axis and Y-axis directions of the image plane coordinate system; $\delta_{lx}$ and $\delta_{ly}$ are the eccentric distortion parameters in the X-axis and Y-axis directions of the image plane coordinate system respectively; $\delta_{bx}$, $\delta_{by}$ are the thin prism distortion parameters in the X-axis and Y-axis directions of the image plane coordinate system respectively; $k_{1}$, $k_{2}$ are the radial distortion parameters; $q_{1}$, $q_{2}$ are eccentric distortion parameters; $s_{1}$, $s_{2}$ are thin prism distortion parameters.

It can be seen from Figure 3 that the vector sum of $\delta_{x}$, $\delta_{y}$ in the image plane coordinate system and $\delta_{pp^{\prime }}$ is the actual translation amount of the image point in the image plane coordinate system:

(23) $$\delta_{pp^{\prime }}=\sqrt{\delta_{x}^{2}+\delta_{y}^{2}}\;.$$

Since UAV vision acquisition systems often use a combination of custom CMOS sensors and fixed-focus lenses, their manufacturing and assembly precision is well guaranteed. Therefore, the calculation of optical distortion displacement $\delta_{pp^{\prime }}$ can be simplified as the combination of first-order radial distortion and second-order eccentric distortion.

(24) $$\delta_{pp^{\prime }}=\sqrt{\begin{array}{c} {\left(k_{1}u_{d}r_{d}^{2}+2q_{1}u_{d}v_{d}+q_{2}\left(r_{d}^{2}+2u_{d}^{2}\right)\right)}^{2}\\ +{\left(k_{1}v_{d}r_{d}^{2}+2q_{2}u_{d}v_{d}+q_{1}\left(r_{d}^{2}+2v_{d}^{2}\right)\right)}^{2} \end{array}}\;.$$

Among them, $u_{d}=x-u_{0}$; $v_{d}=y-v_{0}$; $r_{d}=\sqrt{u_{d}^{2}+v_{d}^{2}}$; $\left(u_{0},v_{0}\right)$ is the coordinate of the main point of the camera.

Obviously, the relationship between the optical distortion displacement $\delta_{pp^{\prime }}$ at any point and its credibility TD conforms to the relationship:

(25) $$TD\propto {\delta_{pp^{\prime }}}^{-1}\;.$$

Based on this conclusion, since two images are captured during each UAV flight (corresponding to two measurement stations), the weighting matrix $W_{1}=\left[\begin{array}{cccc} Q_{11} & & & \\ & Q_{12} & & \\ & & \ldots & \\ & & & Q_{1n} \end{array}\right]$ is constructed according to optical distortion.

(26) $$Q_{1i}=\frac{\sum_{t=1}^{2}{{\delta_{pp^{\prime }}}^{-1}}_{it}}{\sum_{j=1}^{n}\sum_{k=1}^{2}{{\delta_{pp^{\prime }}}^{-1}}_{jk}}\;.$$

Therefore, the weighting matrix based on optical distortion is:

(27) $$W_{1}=\left[\begin{array}{cccc} \frac{\sum_{t=1}^{2}{{\delta_{pp^{\prime }}}^{-1}}_{1t}}{\sum_{j=1}^{n}\sum_{k=1}^{2}{{\delta_{pp^{\prime }}}^{-1}}_{jk}} & \; & & \\ & \frac{\sum_{t=1}^{2}{{\delta_{pp^{\prime }}}^{-1}}_{2t}}{\sum_{j=1}^{n}\sum_{k=1}^{2}{{\delta_{pp^{\prime }}}^{-1}}_{jk}} & \; & \\ & \; & \ldots & \\ & \; & & \frac{\sum_{t=1}^{2}{{\delta_{pp^{\prime }}}^{-1}}_{nt}}{\sum_{j=1}^{n}\sum_{k=1}^{2}{{\delta_{pp^{\prime }}}^{-1}}_{jk}} \end{array}\right]\;.$$

#### 2.2.2. Weighted Algorithm Based on Point Set Rigid Feature Evaluation

The theoretical foundation of the method for computing exterior orientation elements based on station displacement of a rigid point set acknowledges the existence of a rigid point set that is relatively stationary with respect to the photographic center position. It is precisely by treating this point as a perfect rigid body that a series of algorithms based on the object coordinates of various points in the point set, as described earlier, are able to achieve the computation of measurement station exterior orientation elements without calibration.

There is no perfect rigid body in nature. The algorithm described in this article relies on a rigid point set established based on sensors arranged on the UAV. It will theoretically exist. The factors that cause the rigid point set to deviate from the rigid body characteristics mainly include:

(1) The object-space coordinate offset of each point in the rigid point set caused by sensor error.

(2) The displacement of each point of the rigid point set caused by the deformation/vibration of the UAV itself during operation.

(3) The displacement of the camera, lens, and acceleration sensors during the operation of the UAV due to installation reasons.

The characteristics of non-rigidification errors in complex rigid point sets include:

(1) Complexity: The loss of rigid characteristics of the point set is caused by the combined effects of multiple factors.

(2) Randomness: Due to the complexity of contributing factors and the uncertain existence and form of each error, the loss of the rigid characteristics of the point set is random and unpredictable.

(3) Irreparability: Because the loss of rigid characteristics of the rigid point set is complex and random, it is almost impossible to correct through auxiliary data or compensation algorithms.

In order to maximize the accuracy of the solution process, this paper proposes a weighted evaluation based on the quantitative evaluation of the role of each point in the rigid point set used for station displacement solution in the loss of rigid body characteristics of the overall point set. The method uses the component weighting matrix $W_{2}$ to optimize the accuracy of the station displacement solution results.

**Figure 4:**
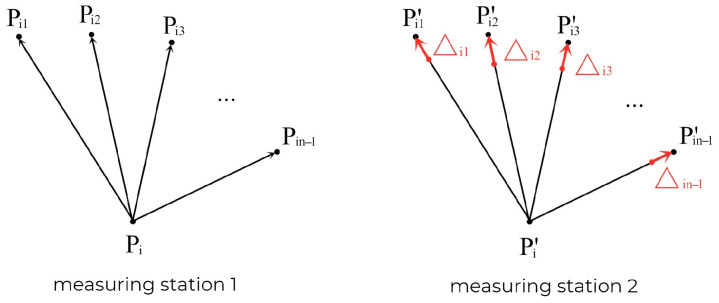
Consistency weighting principle.

As shown in Figure 4, assume that point $P_{i}$ is any point in the rigid point set composed of n points (n≥3). At the positions of measurement station 1 and measurement station 2, due to the loss of rigidity of the point set, there must be a gap between its module and the vector of other points in the point set, that is:

(28) $$\left|\vec{P_{i}P_{ij}}\right|-\left|\vec{P_{i}^{\prime }P_{ij}^{\prime }}\right|=\Delta_{ij}\geq 0,\;j=1,2,\ldots ,n-1,n\;.$$

In order to achieve a quantitative evaluation of the error introduced at point $P_{i}$, similar to when establishing a rigid point set translation algorithm, it is necessary to introduce the concept of an exclusive rigid point set hypothesis:

Definition 2-2: Exclusive rigid point set hypothesis

When performing rigid evaluation on a point to be evaluated, other points in the default rigid point set except this point still form a perfect rigid body.

Based on the above assumptions, the difference between the module of the vector of point $P_{i}$ at each measuring station and other points in the point set and its credibility TD conforms to the relationship:

(29) $$TD\propto \sum_{j=1}^{n-1}{\Delta_{ij}}^{-1}\;.$$

Based on this conclusion, the weighting matrix $W_{2}=\left[\begin{array}{cccc} Q_{21} & & & \\ & Q_{22} & & \\ & & \ldots & \\ & & & Q_{2n} \end{array}\right]$ of the weighting algorithm based on the assessment of the rigidity characteristics of the point set are calculated as follows:

(30) $$\begin{array}{cc} \; & Q_{2i}=\frac{\sum_{j=1}^{n-1}{\Delta_{ij}}^{-1}}{\sum_{k=1}^{n}\sum_{j=1}^{n-1}{\Delta_{kj}}^{-1}}=\frac{\sum_{j=1}^{n-1}{\left\{{\left[A\right]}^{\frac{1}{2}}-{\left[B\right]}^{\frac{1}{2}}\right\}}^{-1}}{\sum_{k=1}^{n}\sum_{j=1}^{n-1}{\left\{{\left[C\right]}^{\frac{1}{2}}-{\left[D\right]}^{\frac{1}{2}}\right\}}^{-1}}\\ \; & A={\left(X_{i}-X_{ij}\right)}^{2}+{\left(Y_{i}-Y_{ij}\right)}^{2}+{\left(Z_{i}-Z_{ij}\right)}^{2}\\ \; & B={\left(X_{i}^{\prime }-X_{ij}^{\prime }\right)}^{2}+{\left(Y_{i}^{\prime }-Y_{ij}^{\prime }\right)}^{2}+{\left(Z_{i}^{\prime }-Z_{ij}^{\prime }\right)}^{2}\\ \; & C={\left(X_{k}-X_{kj}\right)}^{2}+{\left(Y_{k}-Y_{kj}\right)}^{2}+{\left(Z_{k}-Z_{kj}\right)}^{2}\\ \; & D={\left(X_{k}^{\prime }-X_{kj}^{\prime }\right)}^{2}+{\left(Y_{k}^{\prime }-Y_{kj}^{\prime }\right)}^{2}+{\left(Z_{k}^{\prime }-Z_{kj}^{\prime }\right)}^{2}\;. \end{array}$$

$\left(X_{i},Y_{i},Z_{i}\right)$, $\left(X_{ij},Y_{ij},Z_{ij}\right)$, j=1,2,…,n−1,n is the object-space coordinates of each point of the rigid point set at measurement station 1; $\left(X_{i}^{\prime },Y_{i}^{\prime },Z_{i}^{\prime }\right)$, $\left(X_{ij}^{\prime },Y_{ij}^{\prime },Z_{ij}^{\prime }\right)$, j=1,2,…,n−1,n is the object-space coordinates of each point of the rigid point set at 2 measurement stations. We have learned in the uncalibrated solution algorithm of the exterior orientation elements based on the rigid point set displacing station that they are all known quantities.

Therefore, the weighting matrix based on point set rigid feature evaluation is:

(31) $$W_{2}=\left[\begin{array}{cccc} \frac{\sum_{j=1}^{n-1}\Delta_{1j}^{-1}}{\sum_{k=1}^{n}\sum_{j=1}^{n-1}\Delta_{kj}^{-1}} & \; & & \\ & \frac{\sum_{j=1}^{n-1}\Delta_{2j}^{-1}}{\sum_{k=1}^{n}\sum_{j=1}^{n-1}\Delta_{kj}^{-1}} & \; & \\ & \; & \ldots & \\ & \; & & \frac{\sum_{j=1}^{n-1}\Delta_{nj}^{-1}}{\sum_{k=1}^{n}\sum_{j=1}^{n-1}\Delta_{kj}^{-1}} \end{array}\right]\;.$$

#### 2.2.3. Weight Fusion Algorithm

Since there is little experimental data at this stage, it is not enough to support a quantitative assessment of the impact of optical distortion and rigid point set consistency on accuracy. Therefore, before performing weight fusion, we need to make the following assumptions:

Definition 2-3: Affecting the Consistency Assumption

That is to say, it is determined that the effects of optical distortion and rigid point set consistency on accuracy are independent of each other and have equivalent effects.

Based on this assumption, the fusion weight matrix $W=\left[\begin{array}{cccc} Q_{1} & & & \\ & Q_{2} & & \\ & & \ldots & \\ & & & Q_{n} \end{array}\right]$ The calculation method of each element after normalization is:

(32) $$Q_{i}=\frac{Q_{1i}\cdot Q_{2i}}{\sum_{l=1}^{l=n}Q_{1l}\cdot Q_{2l}}=\frac{\frac{\sum_{t=1}^{2}{{\delta_{pp^{\prime }}}^{-1}}_{it}}{\sum_{j=1}^{n}\sum_{k=1}^{2}{{\delta_{pp^{\prime }}}^{-1}}_{jk}}\cdot \frac{\sum_{j=1}^{n-1}{\Delta_{ij}}^{-1}}{\sum_{k=1}^{n}\sum_{j=1}^{n-1}{\Delta_{kj}}^{-1}}}{\sum_{l=1}^{l=n}\frac{\sum_{t=1}^{2}{{\delta_{pp^{\prime }}}^{-1}}_{lt}}{\sum_{j=1}^{n}\sum_{k=1}^{2}{{\delta_{pp^{\prime }}}^{-1}}_{jk}}\cdot \frac{\sum_{j=1}^{n-1}{\Delta_{lj}}^{-1}}{\sum_{k=1}^{n}\sum_{j=1}^{n-1}{\Delta_{kj}}^{-1}}}\;.$$

As shown in Equation (33), based on the above conclusion, we can obtain the fusion weighting matrix W.

(33) $$\begin{array}{cc} W_{1} & =\left[\begin{array}{cccc} \frac{\frac{\sum_{t=1}^{2}{{\delta_{pp^{\prime }}}^{-1}}_{1t}}{\sum_{j=1}^{n}\sum_{k=1}^{2}{{\delta_{pp^{\prime }}}^{-1}}_{jk}}\cdot \frac{\sum_{j=1}^{n-1}{\Delta_{1j}}^{-1}}{\sum_{k=1}^{n}\sum_{j=1}^{n-1}{\Delta_{kj}}^{-1}}}{\sum_{l=1}^{l=n}\frac{\sum_{t=1}^{2}{{\delta_{pp^{\prime }}}^{-1}}_{lt}}{\sum_{j=1}^{n}\sum_{k=1}^{2}{{\delta_{pp^{\prime }}}^{-1}}_{jk}}\cdot \frac{\sum_{j=1}^{n-1}{\Delta_{lj}}^{-1}}{\sum_{k=1}^{n}\sum_{j=1}^{n-1}{\Delta_{kj}}^{-1}}} & \; & & \\ & Q_{2} & \; & \\ & \; & \ldots & \\ & \; & & Q_{n} \end{array}\right]\\ Q_{2} & =\frac{\frac{\sum_{t=1}^{2}{{\delta_{pp^{\prime }}}^{-1}}_{2t}}{\sum_{j=1}^{n}\sum_{k=1}^{2}{{\delta_{pp^{\prime }}}^{-1}}_{jk}}\cdot \frac{\sum_{j=1}^{n-1}{\Delta_{2j}}^{-1}}{\sum_{k=1}^{n}\sum_{j=1}^{n-1}{\Delta_{kj}}^{-1}}}{\sum_{l=1}^{l=n}\frac{\sum_{t=1}^{2}{{\delta_{pp^{\prime }}}^{-1}}_{lt}}{\sum_{j=1}^{n}\sum_{k=1}^{2}{{\delta_{pp^{\prime }}}^{-1}}_{jk}}\cdot \frac{\sum_{j=1}^{n-1}{\Delta_{lj}}^{-1}}{\sum_{k=1}^{n}\sum_{j=1}^{n-1}{\Delta_{kj}}^{-1}}}Q_{n}=\frac{\frac{\sum_{t=1}^{2}{{\delta_{pp^{\prime }}}^{-1}}_{nt}}{\sum_{j=1}^{n}\sum_{k=1}^{2}{{\delta_{pp^{\prime }}}^{-1}}_{jk}}\cdot \frac{\sum_{j=1}^{n-1}{\Delta_{nj}}^{-1}}{\sum_{k=1}^{n}\sum_{j=1}^{n-1}{\Delta_{kj}}^{-1}}}{\sum_{l=1}^{l=n}\frac{\sum_{t=1}^{2}{{\delta_{pp^{\prime }}}^{-1}}_{lt}}{\sum_{j=1}^{n}\sum_{k=1}^{2}{{\delta_{pp^{\prime }}}^{-1}}_{jk}}\cdot \frac{\sum_{j=1}^{n-1}{\Delta_{lj}}^{-1}}{\sum_{k=1}^{n}\sum_{j=1}^{n-1}{\Delta_{kj}}^{-1}}}\;. \end{array}$$

So far, we have provided a comprehensive introduction to the entire process of calculating the exterior orientation elements of the measurement station based on data collected from the UAV’s built-in sensors during flight, along with the corresponding accuracy optimization algorithms.

Next, based on the characteristics of the algorithm, this paper will design numerical simulation verification experiments to to examine the internal consistency of the proposed formulation.

## 3. Numerical Simulation Verification Test

Since the university has not yet procured the UAV platform and related sensors, it is difficult to conduct or fully demonstrate engineering validation experiments based on practical applications. Therefore, the validation was designed at two levels: numerical simulation for model consistency analysis and a controlled indoor bench-scale experiment for physical proof-of-concept verification.

Although numerical simulation cannot replace real-flight validation, numerical simulation verification tests still have positive significance:

(1) Completely implement the exterior orientation elements solution method based on rigid point set resection, and verify the correctness and rationality of the algorithm by comparing input and output parameters;

(2) Through error data simulation, based on the simulated results, we propose recommendations for the required accuracy levels of the sensor group responsible for implementing the various functions of the algorithm in this project.

Since the optical distortion parameters in the numerical simulation analysis process are determined, the weighting algorithm based on optical distortion mentioned in this article cannot be actually implemented. Therefore, it is very regrettable that the error data in this numerical simulation verification test is only designed for the sensor measurement error.

The validation experiment is designed to test high-altitude free-flight trajectory measurement, an extreme measurement scenario, for the following reasons:

(1) Long-distance measurement scenarios provide a better test of the algorithm’s accuracy and correction effectiveness.

(2) High-altitude measurements impose stricter accuracy requirements on sensors, making the results of numerical simulation analysis more meaningful.

The following sections will provide a detailed introduction to this numerical simulation validation experiment.

### 3.1. Test Process

**Figure 5:**
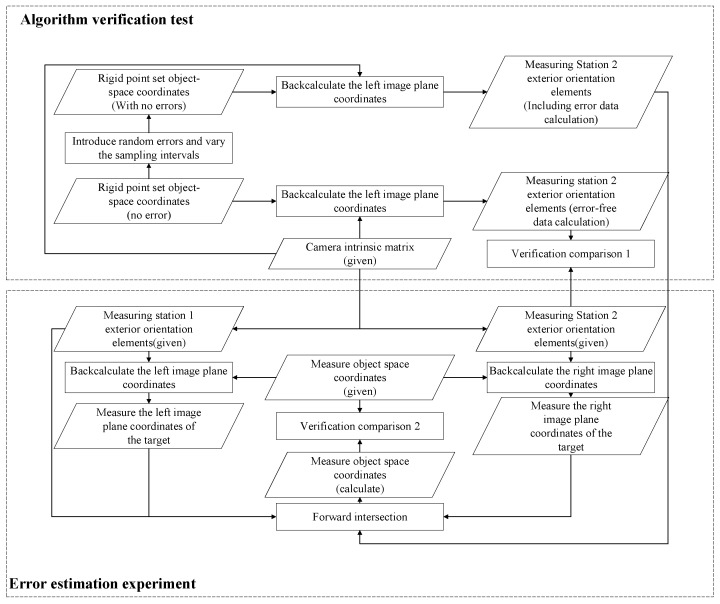
Numerical simulation verification test process.

The process of this test is shown in Figure 5, which is mainly divided into two major components: algorithm verification test and error estimation test:

(1) Algorithm verification test

In this part, this test will be based on the given exterior orientation elements of measurement station 1 and the object-space three-dimensional coordinates of each point of the rigid point set, and use the algorithm described in this article to calculate the exterior orientation elements of measurement station 2 and compare it with the values of the standard external orientation elements given by the measurement station 2 are compared to verify the theoretical correctness of the relevant algorithms.

(2) Error estimation test

(i) Add different levels of random errors to the standard rigid point set object-space three-dimensional coordinates to simulate the random loss of point set rigidity caused by sensor errors and other factors in reality, and solve the problem under this situation. The calculated exterior orientation elements of measuring station 2 are brought into the collinear equation to solve the target object-space coordinates. Finally, by comparing with the standard values, the impact of different levels of sensor errors on the entire solution model is analyzed;

(ii) Change the sampling interval and simulate the impact of different sampling intervals on the measurement baseline. Assess how these changes affect the calculation accuracy to provide information for the subsequent system working modes.

### 3.2. Experimental Software Tools

**Figure 6:**
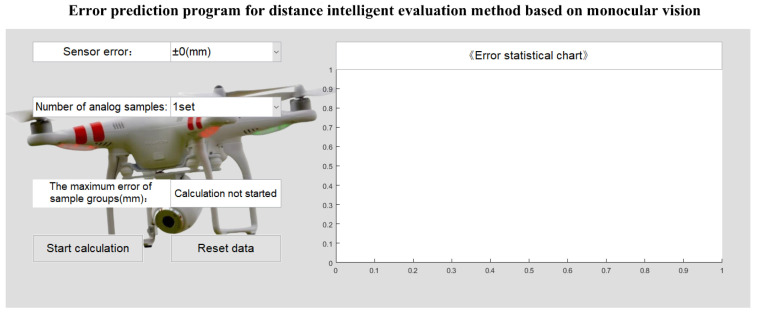
Error prediction program for intelligent distance assessment method based on monocular vision.

As shown in Figure 6, the software used in this experiment is the error prediction program of the intelligent distance assessment method based on monocular vision independently developed by the author of this article. The program’s integrated features include:

(1) Calculation algorithm for exterior orientation elements based on rigid point set resection: This software can directly output the results of resection based on rigid point set, conveniently and quickly verifying the accuracy of external orientation calculations.

(2) Weighting algorithm based on point set rigidity feature evaluation: The software directly introduces a weighting algorithm based on point set rigidity feature evaluation during the settlement process, which can intuitively display the optimized solution accuracy.

(3) Forward intersection calculation based directly on exterior orientation elements: Rear intersection can be bypassed and the impact of exterior orientation elements accuracy on the overall solution accuracy can be directly evaluated.

In addition to the functions mentioned above, practice has proven that this program also significantly increased the execution efficiency of this experiment. Furthermore, this program can serve as a prototype for future UAV-mounted vision measurement systems, using this experiment for preliminary technical validation.

### 3.3. Simulation Parameter Design

As shown in Figure 5, the initial data for this test include: exterior orientation elements of measurement station 1 (given), exterior orientation elements of measurement station 2 (given), rigid point set object-space coordinates (given), camera internal parameter matrix (given), and the measurement target object coordinates (given). Their detailed parameters are as show in Table 1, Table 2, Table 3, Table 4 and Table 5:

Based on the above data, the image-plane coordinates of the object-square coordinates of the target of each measurement in the photographs taken at measuring station 1 and measuring station 2 were calculated as follows (the photographs taken at measuring station 1 were taken as the left slices, and the photographs taken at measuring station 2 were taken as the right slices). As shown in Table 6:

Based on the above parameters and using traditional photographic measurement, the calculated measurement target object-space coordinates (data verification calculation) are shown in Table 7:

Subtracting the data in Table 5 and Table 7, we can get the simulation data verification calculation error shown in Table 8:

As shown in Table 8, based on simulation data, the error level of solution using classic photogrammetry method does not exceed $10^{-9}$ (mm), which proves:

(1) The scientific nature of the simulation data can be used as standard (correct) data to verify the solution accuracy of each algorithm;

(2) The reliability of the simulation data generation program. In the subsequent operation of changing the sampling spacing, it is necessary to generate new rigid point set object-space coordinates and the corresponding exterior orientation elements of the measurement station 2. Since the reliability of the program has been Verify that the text structure is reasonable and compact. In the future, we will directly quote the data automatically generated by the program as the calculation and comparison benchmark, and will no longer conduct verification as in this chapter.

### 3.4. Algorithm Verification Test Results

According to the algorithm described in Section 2 the intermediate data for solving the external orientation elements based on the rigid point set resection, namely the rotation matrix and translation vector, are:

(1) Rotation matrix:

$R_{S}=\left[\begin{array}{ccc} 0.991711176477632 & -0.078202201739515 & 0.101947820439882\\ 0.084006923422541 & 0.995004165278027 & -0.053940225216950\\ -0.097220261604409 & 0.062057446954158 & 0.993326277721023 \end{array}\right]$

(2) Translation vector: $S_{S}=\left[\begin{array}{c} 0.000000\\ 0.000000\\ -100000 \end{array}\right]$

The exterior orientation elements of measurement station 2 calculated from this are:

Subtracting the data in Table 2 and Table 9, we can get the simulation data verification calculation error shown in Table 10:

### 3.5. Error Estimation Test Results

As mentioned above, in this numerical simulation analysis test, the specific influencing factors are applied in the following ways:

(1) Sampling spacing: achieved by adjusting the measurement station exterior orientation elements’ line elements;

(2) Sensor error: achieved by applying random errors to the object-space coordinates of the rigid point set.

**Figure 7:**
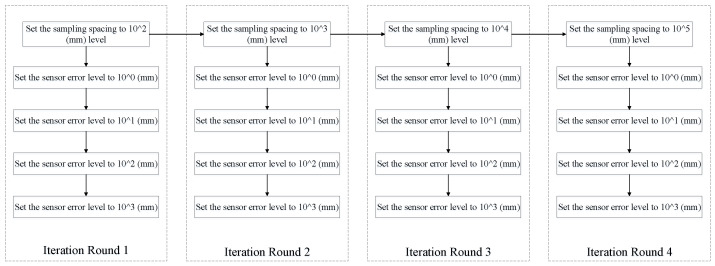
Simulation error application process.

During the test, the specific simulation error application process is shown in Figure 7:

#### 3.5.1. Iteration Round 1 Data

**Figure 8:**
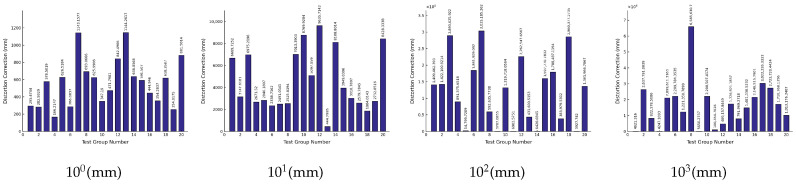
Error in calculating the object-space coordinates of the measurement target in iteration round 1.

In iteration round 1, the exterior orientation elements of each measurement station and the corresponding rigid point set object-space coordinates data are as show in Table 11:

Based on the above data, the iteration results corresponding to the solution errors of the object-space coordinates of each level of measurement target are as shown in Figure 8. The numbers of each test point of the simulated measurement target are the same as Table 5:

#### 3.5.2. Iteration Round 2 Data

**Figure 9:**
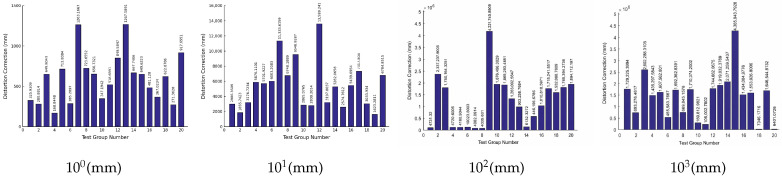
Error in calculating the object-space coordinates of the measurement target in iteration round 2.

In iteration round 2, the exterior orientation elements of each measurement station and the corresponding rigid point set object-space coordinates data are as show in Table 12:

Based on the above data, the iteration results corresponding to the solution errors of the object-space coordinates of each level of measurement target are as shown in Figure 9. The numbers of each test point of the simulated measurement target are the same as Table 5:

#### 3.5.3. Iteration Round 3 Data

**Figure 10:**
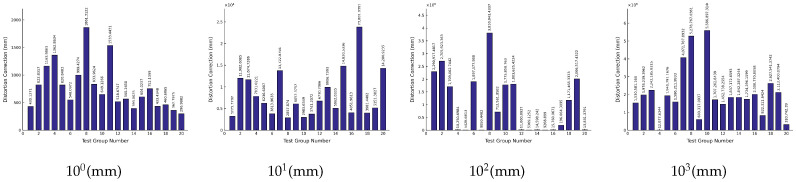
Error in calculating the object-space coordinates of the measurement target in iteration round 3.

In iteration round 3, the exterior orientation elements of each measurement station and the corresponding rigid point set object-space coordinates data are as show in Table 13:

Based on the above data, the iteration results corresponding to the solution errors of the object-space coordinates of each level of measurement target are as shown in Figure 10. The numbers of each test point of the simulated measurement target are the same as Table 5:

#### 3.5.4. Iteration Round 4 Data

**Figure 11:**
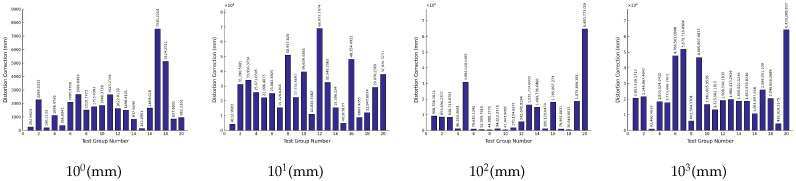
Error in calculating the object-space coordinates of the measurement target in iteration round 4.

In iteration round 4, the exterior orientation elements of each measurement station and the corresponding rigid point set object-space coordinates data are as show in Table 14:

Based on the above data, the iteration results corresponding to the solution errors of the object-space coordinates of each level of measurement target are as shown in Figure 11. The numbers of each test point of the simulated measurement target are the same as Table 5:

### 3.6. Test Result Analysis

#### 3.6.1. Data Correctness Proof

In order to prove the correctness of each round of iteration parameters, in this experiment, it can be achieved by setting the random error level to “0 (mm)”. Under this setting, the entire solution process will be based on absolutely correct parameters. Solution, the solution error of the object coordinates of each attack target should be close to zero.

**Figure 12:**
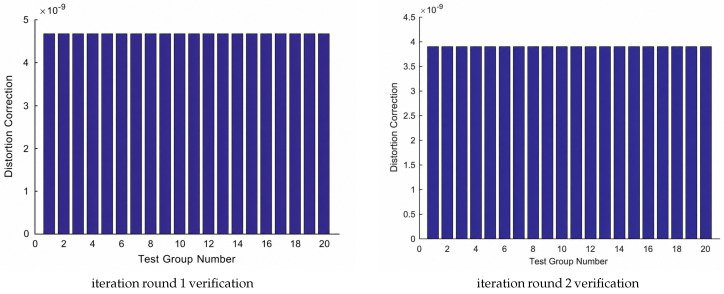

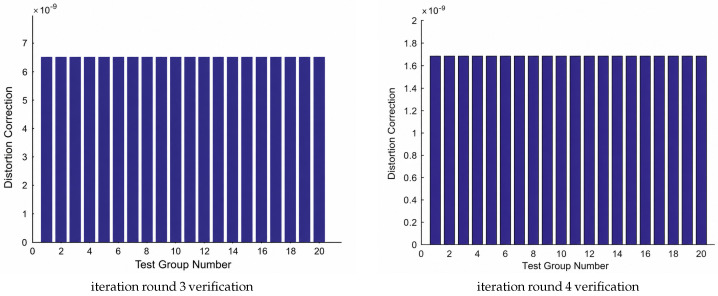
Data correctness check results.

**Figure 13:**
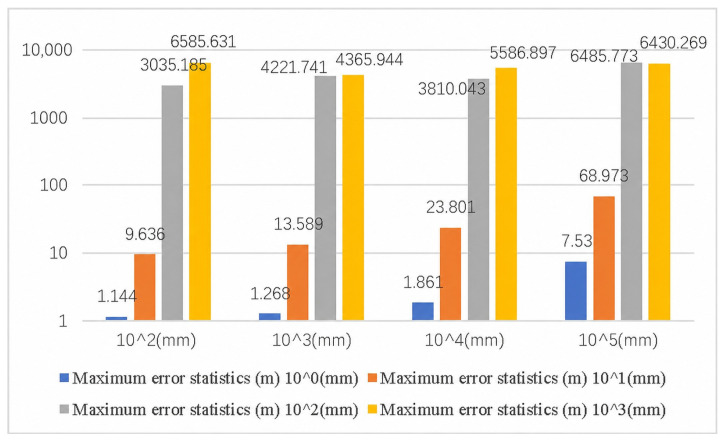
Iteration error statistics plot.

Based on this principle, the verification iteration results of each round are as shown in Figure 12:

It can be seen from Figure 13 that the verification error level of each round of iteration does not exceed the $10^{-9}$ (mm) level, so the synthetic data and iteration results are numerically consistent within the designed simulation setting.

#### 3.6.2. Iteration Error Statistics

As show in Table 15. To more accurately evaluate the impact of the systematic error of the proposed method on UAV navigation accuracy under different iteration conditions, the maximum calculation error of the measured object-space coordinates was selected for each iteration cycle in the statistical analysis.

Table 16 and Table 17 present the mean error statistics and root-mean-square error (RMSE) statistics under different iteration conditions.

The mean error and RMSE statistics indicate that, compared with using only the maximum error as the evaluation metric, the mean error and RMSE provide a more comprehensive representation of the overall error level of the algorithm under different sampling intervals and sensor error levels. Overall, as the sensor error level increased from $10^{0}$ mm to $10^{3}$ mm, both statistical indicators showed a clear increasing trend, indicating that the coordinate accuracy of the rigid point set is an important factor affecting exterior orientation propagation and subsequent target-point reconstruction.

It should be noted that the numerical simulation and the controlled bench-top experiment are designed for different validation purposes and should not be directly compared in terms of absolute error magnitude. The numerical simulation intentionally adopts large sampling intervals and large rigid-point coordinate errors as stress-test conditions to examine the theoretical response and sensitivity of the proposed station-displacement model under extreme assumptions. Therefore, the kilometer-level errors observed in some simulation cases reflect the behavior of the model under intentionally amplified error inputs, rather than the expected accuracy in a realistic measurement scenario. In contrast, the bench-top experiment is conducted at a more realistic physical scale using real imaging data and total-station reference measurements. Its purpose is to verify whether the complete processing chain can be implemented under controlled physical conditions. Therefore, these two validation approaches are complementary rather than directly comparable: the numerical simulation evaluates the sensitivity and theoretical limits of the model, whereas the bench-top experiment provides physical proof-of-concept evidence at a realistic measurement scale.

#### 3.6.3. Result Analysis

(1) Analysis of algorithm verification test results:

As shown in Table 10, the calculation error of the exterior orientation elements of measurement station 2 obtained based on the rigid point set displacing solution is at the level of $10^{-6}$ (mm) and $10^{-6}$ (rad) The errors are all 0, so the simulation results indicate that the implementation is consistent under ideal synthetic inputs.

(2) Analysis of error estimation test results:

The results after visualization of each parameter in Table 15 are shown in Figure 13:

From Figure 13 we can draw the following conclusions:

(1) The sampling spacing has an impact on the overall measurement accuracy of the system, and the sampling spacing is not the bigger the better or the smaller the better, but there is an optimal intermediate value;

(2) The accuracy of the sensors (the accuracy of the object space coordinates of each point in the rigid point set) has a significant impact on the overall measurement accuracy of the system. Under the current simulation settings, the results preliminarily suggest that rigid-point coordinate errors at approximately the 10 mm level may serve as a reference range for the input accuracy of the proposed model. However, this value should be interpreted only as a modeling-based preliminary estimate rather than as a confirmed requirement for sensor accuracy, because the bench-top experiment uses total-station measurements as reference inputs and the present study has not yet performed real-flight sensor-error validation.

## 4. Bench-Top Proof-of-Concept Experiment

To further evaluate the implementability of the proposed method using real imaging devices under realistic measurement conditions, an indoor controlled bench-top proof-of-concept experimental platform was constructed on the basis of the numerical simulation analysis. This experiment is not intended to replace validation through actual UAV flight tests; rather, it aims to provide a proof-of-concept assessment of the feasibility of the proposed method in a real physical scenario.

**Figure 14:**
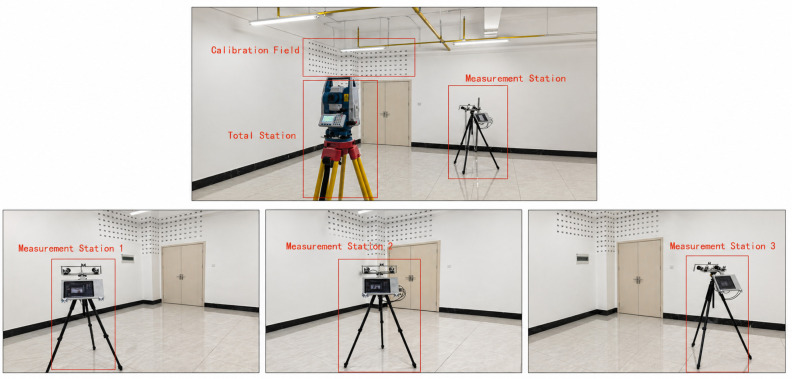
Experimental Site Layout.

AS shown in Figure 14. The experimental procedure in this section is as follows.

(1) First, the validation experiment was conducted using a wall-mounted calibration field, together with a rigid point set mounted on a simulated measurement device, to examine the station-displacement algorithm. The calibration of the control field and the measurement of the rigid point set at each measurement station were both performed using a Tianyu CTS-630 total station in reflectorless mode. When operating in reflectorless mode within a distance of 10 m, the maximum measurement error of the total station is ±1 mm.

(2) Second, the vision measurement system was sequentially placed at measurement station 1, measurement station 2, and measurement station 3 for image acquisition. To maintain consistency with the monocular free-flight photogrammetry framework established in this study, only the images captured by the left camera of the binocular system were used for computation. The exterior orientation elements of measurement station 1 were determined by resection using the wall-mounted control points, whereas those of measurement stations 2 and 3 were calculated using the rigid point set station-displacement algorithm proposed in this paper.

(3) Subsequently, the left-camera image acquired at measurement station 1 was paired with the left-camera images acquired at measurement stations 2 and 3, respectively, forming two sets of cross-station monocular stereo image pairs. In each image pair, the image from measurement station 1 was used as the left image, while the image from measurement station 2 or measurement station 3 was used as the right image.

(4) After the two cross-station image pairs were obtained, the image coordinates of the target points to be measured were extracted from the left and right images. The object-space three-dimensional coordinates of each target point were then calculated based on the collinearity equations and forward intersection.

(5) Finally, the three-dimensional coordinates of the target points measured by the total station were used as reference values. The measurement results were evaluated from three perspectives: coordinate component errors, single-point three-dimensional positioning errors, and spatial distance errors between feature-point pairs.

The experiment was carried out in an indoor controlled environment. A regular target control field was arranged on the wall, with a center-to-center spacing of 150 mm between adjacent targets in both the horizontal and vertical directions.

In this experiment, the total-station measurement of the rigid point set at each measurement station was used to simulate the spatial displacement measurement that would be performed by accelerometers during actual operation. Since the two measurement approaches are of comparable accuracy levels, the experimental design is considered scientifically reasonable.

### 4.1. Experimental Data Acquisition

The data acquired and used in the bench-top experiment mainly consisted of five categories. First, the exterior orientation elements of measurement station 1 were determined by resection using the wall-mounted control field. Second, the exterior orientation elements of measurement stations 2 and 3 were calculated using the rigid point set station-displacement algorithm. Third, the object-space coordinates of five rigid-point targets at the three measurement stations were measured using the total station and used as input data for the station-displacement algorithm. Fourth, the camera intrinsic parameters were used, including the focal length, pixel size, and principal point coordinates. Fifth, the reference coordinates of the target points measured by the total station and their image coordinates in the photographs acquired at different measurement stations were collected. As show in Table 18, Table 19, Table 20, Table 21 and Table 22. Together, these data constituted the computational basis for the bench-top proof-of-concept experiment in this study.

**Figure 15:**
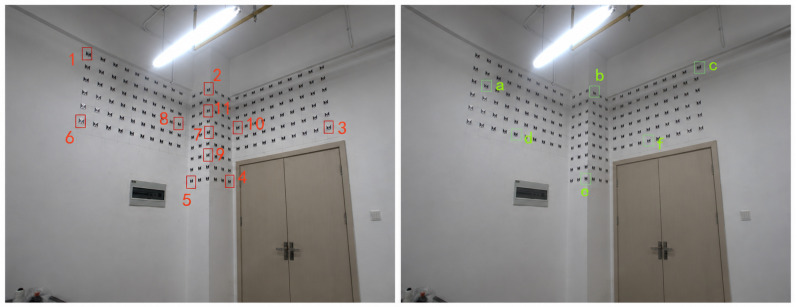
Control Point Set and Measurement Point Set.

As shown in Figure 15, the left image shows the control point set used to determine the exterior orientation elements of measurement station 1, while the right image shows the measurement point set used in this experiment.

The data collected in the experiment are shown in the Table 23, Table 24, Table 25, Table 26 and Table 27.

Based on the above parameters, the object-space coordinates of the target points were calculated using the classical photogrammetric method, as shown in the table.

### 4.2. Analysis of Experimental Results

To quantitatively evaluate the consistency between the bench-top experimental results and the reference values measured by the total station, error analysis was conducted at three levels. First, the coordinate component errors of each target point in the X, Y, and Z directions were calculated. Second, the single-point three-dimensional positioning error was computed to characterize the spatial deviation between the point coordinates obtained by forward intersection and the corresponding reference coordinates. Finally, the spatial distance errors between feature-point pairs were calculated to evaluate the ability of the measurement results to preserve local geometric relationships.

(34) $$\Delta X=X_{m}-X_{r},\;\Delta Y=Y_{m}-Y_{r},\;\Delta Z=Z_{m}-Z_{r}$$

(35) $$\Delta S=\sqrt{{\left(\Delta X\right)}^{2}+{\left(\Delta Y\right)}^{2}+{\left(\Delta Z\right)}^{2}}$$

In the equations, $X_{m},Y_{m},Z_{m}$ denote the measured coordinates obtained by forward intersection, whereas $X_{r},Y_{r},Z_{r}$ denote the reference coordinates measured by the total station. On this basis, the magnitude of the coordinate error vector was further used to represent the single-point three-dimensional spatial error. The calculation results are shown in the Table 28 and Table 29.

**Figure 16:**
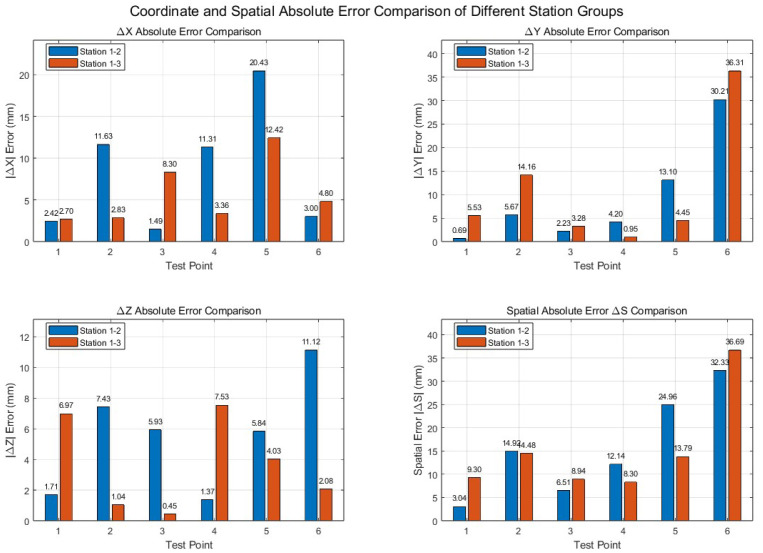
Measurement Error Statistics for Target Points.

As shown in Figure 16, For the image pair formed by measurement stations 1 and 2, the mean three-dimensional positioning error was 15.652 mm. For the image pair formed by measurement stations 1 and 3, the mean three-dimensional positioning error was 15.249 mm. The overall mean three-dimensional positioning error of the two image pairs was 15.450 mm, and the maximum three-dimensional positioning error was 36.685 mm.

**Figure 17:**
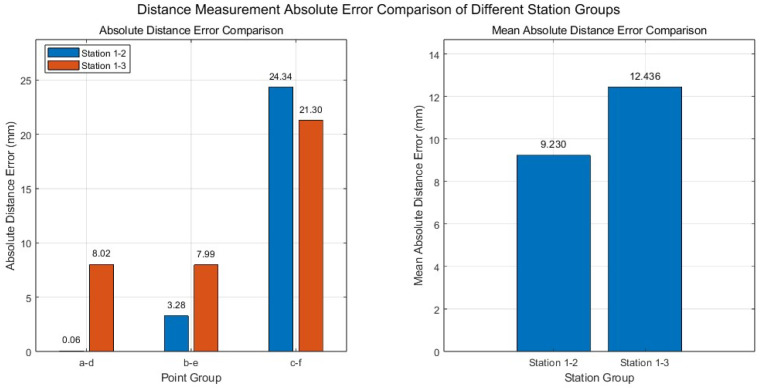
Spatial Distance Error Statistics for Target-Point Groups.

As shown in Figure 17. Furthermore, to evaluate the ability of the measurement results to preserve local spatial geometric relationships, three feature-point pairs, namely a–d, b–e and c–f, were selected for spatial distance comparison. For the image pair formed by measurement stations 1 and 2, the distance errors of the three feature-point pairs were 0.064 mm, 3.284 mm and −24.341 mm, respectively, with a mean absolute distance error of 9.230 mm. For the image pair formed by measurement stations 1 and 3, the distance errors of the three feature-point pairs were −8.020 mm, −7.987 mm, and −21.302 mm, respectively, with a mean absolute distance error of 12.436 mm.

Taken together, these results indicate that the proposed rigid point set station-displacement method for exterior orientation computation can complete the full workflow in a real indoor bench-top environment, including the establishment of the exterior orientation of the initial measurement station, the computation of the exterior orientation of subsequent measurement stations, and the forward-intersection-based measurement of target points. The experimental results demonstrate the clear feasibility of the proposed method in a controlled physical scenario.

Therefore, the results of this experiment are positioned as a proof-of-concept validation of the proposed free-flight photogrammetry framework, rather than as definitive evidence of its engineering performance under real flight conditions.

## 5. Conclusions

This study addresses the problem of acquiring exterior orientation elements in free-flight photogrammetry under control-point-free conditions and proposes a proof-of-concept framework based on the fusion of monocular vision and sensor-group displacement information. The framework is centered on rigid point set station displacement. By using changes in the spatial relationship of the rigid point set between adjacent measurement stations, the exterior orientation elements of subsequent stations can be calculated, thereby reducing the reliance on repeated resection using ground control points in multi-station photogrammetry. This provides a technically feasible research direction for multi-station monocular photogrammetry under free-flight conditions.

At the methodological level, this study establishes an exterior orientation computation model based on rigid point set station displacement and introduces a composite weighting strategy that combines the influence of optical distortion with the consistency evaluation of the rigid point set. This strategy is used to improve the rationality of point-set information utilization during station-displacement computation. At the validation level, numerical simulations were first conducted to analyze the effects of different sampling intervals and rigid-point coordinate errors on exterior orientation computation and target-point reconstruction. The results indicate that the coordinate accuracy of the rigid points is an important factor affecting the computational results of the proposed method.

To address the limitations of purely numerical simulation-based validation, an indoor controlled bench-top proof-of-concept experiment was further constructed. The experimental results show that the overall mean three-dimensional positioning error of the two cross-station image pairs was 15.450 mm, and the maximum three-dimensional positioning error was 36.685 mm. The mean absolute distance errors for station 1–station 2 and station 1–station 3 were 9.230 mm and 12.436 mm, respectively. These results indicate that the proposed framework can complete inter-station exterior orientation computation and three-dimensional target-point measurement in a controlled indoor physical scenario, demonstrating clear proof-of-concept significance.

It should be noted that the current work remains at the proof-of-concept stage and does not constitute a complete validation under real UAV flight conditions. Therefore, the results of this study should be understood as a preliminary verification of the theoretical and physical feasibility of free-flight photogrammetry. Future work will further improve the error propagation model and failure criteria, and real flight experiments will be conducted to evaluate the applicability and robustness of the proposed framework in complex field environments. 

## Figures and Tables

**Table 1:** Measurement Station 1 Exterior Orientation Elements (given).

Line Elements			Angular Elements		
X1 (mm)	Y1 (mm)	Z1 (mm)	$\varphi_{1}$ (rad)	$\omega_{1}$ (rad)	$\kappa_{1}$ (rad)
1,000,000	1,000,000	1,000,000	1	1	1

**Table 2:** Measurement Station 2 Exterior Orientation Elements (given).

Line Elements			Angular Elements		
X2 (mm)	Y2 (mm)	Z2 (mm)	$\varphi_{2}$ (rad)	$\omega_{2}$ (rad)	$\kappa_{2}$ (rad)
1,015,456.795	1,025,070.863	858,163.463	0.900000	1.100000	1.00000

**Table 3:** Rigid Point Set Object-Space Coordinates (given).

Test Point Number		X (mm)	Y (mm)	Z (mm)
Measurement station 1	A1	100	0	0
	B1	0	100	0
	C1	0	0	100
	D1	100	100	100
Measurement station 2	A2	99.17111765	8.400692342	−100,009.722
	B2	−7.820220174	99.50041653	−99,993.79426
	C2	10.19478204	−5.394022522	−99,900.66737
	D2	101.5456795	102.5070863	−99,904.18365

**Table 4:** Camera Internal Parameter Matrix (given).

Camera Parameters	
FJ (mm)	50
pixel (µm)	3.76
Ox (pixel)	4752
Oy (pixel)	3168

**Table 5:** Measurement Target Object Coordinates (given).

Test Point Number	X (mm)	Y (mm)	Z (mm)
1	0	0	0
2	1000	0	0
3	0	1000	0
4	0	0	1000
5	5000	0	0
6	0	5000	0
7	0	0	5000
8	10,000	0	0
9	0	10,000	0
10	0	0	10,000
11	10,000	10,000	10,000

**Table 6:** Measurement Target Image Plane Coordinates (calculation).

Serial Number	xl	yl	xr	yr
1	49.192454	−50.175683	43.541483	−47.062617
2	49.229874	−50.156705	43.568662	−47.035205
3	49.211053	−50.232311	43.560601	−47.115159
4	49.136483	−50.138082	43.486531	−47.032345
5	49.379895	−50.080621	43.677566	−46.925365
6	49.285763	−50.459775	43.637404	−47.326236
7	48.912926	−49.987900	43.267026	−46.911422
8	49.568188	−49.985127	43.814120	−46.787639
9	49.379861	−50.746269	43.734160	−47.592150
10	48.634207	−49.800661	42.993316	−46.760638
11	49.192454	−50.175683	43.453712	−47.010254

**Table 7:** Measurement Target Object-Space Coordinates (Data Verification Calculation).

Test Site Number	X (mm)	Y (mm)	Z (mm)
1	−4.62752 $\times \;10^{-11}$	−2.1631 $\times \;10^{-10}$	−6.81256 $\times \;10^{-11}$
2	1000	−1.38582 $\times \;10^{-9}$	−1.42284 $\times \;10^{-9}$
3	−1.07148 $\times \;10^{-9}$	1000	−8.33166 $\times \;10^{-10}$
4	1.09382 $\times \;10^{-9}$	8.87668 $\times \;10^{-10}$	1000
5	5000	−2.53657 $\times \;10^{-9}$	−2.20078 $\times \;10^{-9}$
6	1.07221 $\times \;10^{-9}$	5000	1.01806 $\times \;10^{-9}$
7	5.88274 $\times \;10^{-10}$	4.722 $\times \;10^{-10}$	5000
8	10,000	5.08974 $\times \;10^{-10}$	6.15758 $\times \;10^{-10}$
9	−4.04323 $\times \;10^{-10}$	10,000	−4.44361 $\times \;10^{-10}$
10	4.88792 $\times \;10^{-10}$	2.57976 $\times \;10^{-10}$	10,000
11	10,000	10,000	10,000

**Table 8:** Simulated Data Verification Calculation Error.

Test Site Number	△X (mm)	△Y (mm)	△Z (mm)
1	4.62752 $\times \;10^{-11}$	2.1631 $\times \;10^{-10}$	6.81256 $\times \;10^{-11}$
2	1.52147 $\times \;10^{-9}$	1.38582 $\times \;10^{-9}$	1.42284 $\times \;10^{-9}$
3	1.07148 $\times \;10^{-9}$	1.24999 $\times \;10^{-9}$	8.33166 $\times \;10^{-10}$
4	−1.09382 $\times \;10^{-9}$	−8.87668 $\times \;10^{-10}$	−9.94419 $\times \;10^{-10}$
5	2.44654 $\times \;10^{-9}$	2.53657 $\times \;10^{-9}$	2.20078 $\times \;10^{-9}$
6	−1.07221 $\times \;10^{-9}$	−1.07866 $\times \;10^{-9}$	−1.01806 $\times \;10^{-9}$
7	−5.88274 $\times \;10^{-10}$	−4.722 $\times \;10^{-10}$	−6.72117 $\times \;10^{-10}$
8	−5.29326 $\times \;10^{-10}$	−5.08974 $\times \;10^{-10}$	−6.15758 $\times \;10^{-10}$
9	4.04323 $\times \;10^{-10}$	4.32919 $\times \;10^{-10}$	4.44361 $\times \;10^{-10}$
10	−4.88792 $\times \;10^{-10}$	−2.57976 $\times \;10^{-10}$	−5.00222 $\times \;10^{-10}$
11	−1.57706 $\times \;10^{-9}$	−1.44428 $\times \;10^{-9}$	−1.39335 $\times \;10^{-9}$

**Table 9:** Measurement Station 2 Exterior Orientation Elements (calculation).

Line Elements			Angular Elements		
X2 (mm)	Y2 (mm)	Z2 (mm)	$\varphi_{2}$ (rad)	$\omega_{2}$ (rad)	$\kappa_{2}$ (rad)
1,015,456.795	1,025,070.863	858,163.463	0.900000	1.100000	1.000000

**Table 10:** Calculation Error of Exterior Orientation Elements of Measurement Station 2.

Line Element Error			Angular Element Error		
△X (mm)	△Y (mm)	△Z (mm)	△φ (rad)	△ω (rad)	△κ (rad)
0.000000	0.000000	0.000000	0.000000	0.000000	0.000000

**Table 11:** Iteration Round 1 Related Parameters.

Parameter Name		Parameter Value		
Measurement station 1 exterior orientation elements	Line elements	X1 (mm)	Y1 (mm)	Z1 (mm)
		1,000,000.000	1,000,000.000	1,000,000.000
	Angular elements	φ1 (rad)	ω1 (rad)	κ1 (rad)
		1.000	1.000	1.000
Measurement station 2 exterior orientation elements	Line elements	X1 (mm)	Y1 (mm)	Z1 (mm)
		1,015,456.795	1,025,070.863	958,063.463
	Angular elements	φ1 (rad)	ω1 (rad)	κ1 (rad)
		0.900	1.100	1.000
Measurement station 1 rigid point set	Rigid point number	X	Y	Z
	1	100.000	0.000	0.000
	2	0.000	100.000	0.000
	3	0.000	0.000	100.000
	4	100.000	100.000	100.000
Measurement station 2 rigid point set	Rigid point number	X	Y	Z
	1	99.171	8.401	−109.722
	2	−7.820	99.500	−93.794
	3	10.195	−5.394	−0.667
	4	101.546	102.507	−4.184

**Table 12:** Iteration Round 2 Related Parameters.

Parameter Name		Parameter Value		
Measurement station 1 exterior orientation elements	Line elements	X1 (mm)	Y1 (mm)	Z1 (mm)
		1,000,000.000	1,000,000.000	1,000,000.000
	Angular elements	φ1 (rad)	ω1 (rad)	κ1 (rad)
		1.000	1.000	1.000
Measurement station 2 exterior orientation elements	Line elements	X1 (mm)	Y1 (mm)	Z1 (mm)
		1,015,456.795	1,025,070.863	957,163.463
	Angular elements	φ1 (rad)	ω1 (rad)	κ1 (rad)
		0.900	1.100	1.000
Measurement station 1 rigid point set	Rigid point number	X	Y	Z
	1	100.000	0.000	0.000
	2	0.000	100.000	0.000
	3	0.000	0.000	100.000
	4	100.000	100.000	100.000
Measurement station 2 rigid point set	Rigid point number	X	Y	Z
	1	99.171	8.401	−1009.722
	2	−7.820	99.500	−993.794
	3	10.195	−5.394	−900.667
	4	101.546	102.507	−904.184

**Table 13:** Iteration Round 3 Related Parameters.

Parameter Name		Parameter Value		
Measurement station 1 exterior orientation elements	Line elements	X1 (mm)	Y1 (mm)	Z1 (mm)
		1,000,000.000	1,000,000.000	1,000,000.000
	Angular elements	φ1 (rad)	ω1 (rad)	κ1 (rad)
		1.000	1.000	1.000
Measurement station 2 exterior orientation elements	Line elements	X1 (mm)	Y1 (mm)	Z1 (mm)
		1,015,456.795	1,025,070.863	948,163.463
	Angular elements	φ1 (rad)	ω1 (rad)	κ1 (rad)
		0.900	1.100	1.000
Measurement station 1 rigid point set	Rigid point number	X	Y	Z
	1	100.000	0.000	0.000
	2	0.000	100.000	0.000
	3	0.000	0.000	100.000
	4	100.000	100.000	100.000
Measurement station 2 rigid point set	Rigid point number	X	Y	Z
	1	99.171	8.401	−10,009.722
	2	−7.820	99.500	−9993.794
	3	10.195	−5.394	−9900.667
	4	101.546	102.507	−9904.184

**Table 14:** Iteration Round 4 Related Parameters.

Parameter Name		Parameter Value		
Measurement station 1 exterior orientation elements	Line elements	X1 (mm)	Y1 (mm)	Z1 (mm)
		1,000,000.000	1,000,000.000	1,000,000.000
	Angular elements	φ1 (rad)	ω1 (rad)	κ1 (rad)
		1.000	1.000	1.000
Measurement station 2 exterior orientation elements	Line elements	X1 (mm)	Y1 (mm)	Z1 (mm)
		1,015,456.795	1,025,070.863	858,163.463
	Angular elements	φ1 (rad)	ω1 (rad)	κ1 (rad)
		0.900	1.100	1.000
Measurement station 1 rigid point set	Rigid point number	X	Y	Z
	1	100.000	0.000	0.000
	2	0.000	100.000	0.000
	3	0.000	0.000	100.000
	4	100.000	100.000	100.000
Measurement station 2 rigid point set	Rigid point number	X	Y	Z
	1	99.171	8.401	−100,009.722
	2	−7.820	99.500	−99,993.794
	3	10.195	−5.394	−99,900.667
	4	101.546	102.507	−99,904.184

**Table 15:** Iteration Error Statistics.

Sampling Interval	Maximum Error Statistics (m)			
	$10^{0}$ (mm)	$10^{1}$ (mm)	$10^{2}$ (mm)	$10^{3}$ (mm)
$10^{2}$ (mm)	1.144	9.636	3035.185	6585.631
$10^{3}$ (mm)	1.268	13.589	4221.741	4365.944
$10^{4}$ (mm)	1.861	23.801	3810.043	5586.897
$10^{5}$ (mm)	7.530	68.973	6485.773	6430.269

**Table 16:** Mean Error Statistics.

Sampling Interval	$10^{0}$ (mm)	$10^{1}$ (mm)	$10^{2}$ (mm)	$10^{3}$ (mm)
$10^{2}$ (mm)	0.559	4.564	1206.253	1678.572
$10^{3}$ (mm)	0.597	5.405	1131.836	1380.853
$10^{4}$ (mm)	0.777	7.991	1016.304	2063.745
$10^{5}$ (mm)	1.818	26.940	996.025	2351.451

**Table 17:** Root Mean Square Error Statistics.

Sampling Interval	$10^{0}$ (mm)	$10^{1}$ (mm)	$10^{2}$ (mm)	$10^{3}$ (mm)
$10^{2}$ (mm)	0.622	5.295	1566.895	2249.144
$10^{3}$ (mm)	0.670	6.238	1565.281	1712.059
$10^{4}$ (mm)	0.879	9.599	1516.077	2491.970
$10^{5}$ (mm)	2.495	31.489	1802.181	2831.735

**Table 18:** Object-Space Coordinates of Rigid Points at Each Measurement Station.

	Rigid Point Set	X (mm)	Y (mm)	Z (mm)
Measurement Station 1	1	2345	1766	−174
	2	2337	1524	−240
	3	2149	1793	−242
	4	2129	1369	−549
	5	1933	1647	−558
Measurement Station 2	1	2643	3163	−179
	2	2537	2944	−242
	3	2473	3266	−245
	4	2280	2886	−547
	5	2212	3218	−556
Measurement Station 3	1	2791	4153	−179
	2	2640	3958	−243
	3	2645	4287	−245
	4	2376	3956	−548
	5	2382	4300	−557

**Table 19:** Exterior Orientation Elements of Measurement Station 1 (Calculated by Resection).

Linear Elements			Angular Elements		
X1 (mm)	Y1 (mm)	Z1 (mm)	φ1 (rad)	ω1 (rad)	κ1 (rad)
2392.735	613.8785	−393.542	−1.866	3.509	−22.093

**Table 20:** Exterior Orientation Elements of Measurement Station 2 (Calculated by Station Displacement).

Linear Elements			Angular Elements		
X2 (mm)	Y2 (mm)	Z2 (mm)	φ2 (rad)	ω2 (rad)	κ2 (rad)
2219.673	2088.211	−387.904	1.175	−0.761	−0.260

**Table 21:** Exterior Orientation Elements of Measurement Station 3 (Calculated by Station Displacement).

Linear Elements			Angular Elements		
X3 (mm)	Y3 (mm)	Z3 (mm)	φ3 (rad)	ω3 (rad)	κ3 (rad)
2148.018	3191.284	−391.025	1.066	−0.953	−0.405

**Table 22:** Camera Intrinsic Parameter Matrix.

Camera Parameters	
FJ (mm)	8
pixel (µm)	3.45
Ox (pixel)	4096
Oy (pixel)	2460

**Table 23:** Object-Space Coordinates of Target Points Measured by the Total Station.

No.	X (mm)	Y (mm)	Z (mm)
a	−288	3145	1175
b	−738	3152	724
c	−1681	2809	270
d	−1806	2753	1472
e	−2188	2068	723
f	−2211	1300	1473

**Table 24:** Image-Plane Coordinates of Target Points at Measurement Stations 1 and 2.

No.	xl (pixel)	yl (pixel)	xr (pixel)	yr (pixel)
a	1124	823	1077	712
b	1833	961	2072	763
c	2606	940	3005	551
d	1288	1209	1345	1106
e	1760	1571	1994	1474
f	2233	1350	2576	1159

**Table 25:** Image-Plane Coordinates of Target Points at Measurement Stations 1 and 3.

No.	xl (pixel)	yl (pixel)	xr (pixel)	yr (pixel)
a	1124	823	858	753
b	1833	961	1934	713
c	2606	940	2823	358
d	1288	1209	1147	1114
e	1760	1571	1831	1457
f	2233	1350	2425	1090

**Table 26:** Object-Space Coordinates of Target Points Calculated from Measurement Stations 1 and 2.

No.	X (mm)	Y (mm)	Z (mm)
a	−285.579	3144.306	1176.707
b	−726.372	3146.328	716.566
c	−1679.511	2806.773	264.067
d	−1794.691	2757.201	1470.631
e	−2208.435	2081.096	728.839
f	−2207.996	1330.207	1461.876

**Table 27:** Object-Space Coordinates of Target Points Calculated from Measurement Stations 1 and 3.

No.	X (mm)	Y (mm)	Z (mm)
a	−290.704	3150.530	1168.025
b	−735.166	3137.837	722.955
c	−1689.297	2812.284	269.550
d	−1802.644	2752.052	1464.468
e	−2200.417	2072.447	718.973
f	−2215.803	1336.310	1470.923

**Table 28:** Calculation Errors of Coordinates Solved from Different Measurement Station Groups.

No.	△X (mm)	△Y (mm)	△Z (mm)	△S (mm)
Station 1– Station 2	2.421	−0.694	1.707	3.043
	11.628	−5.672	−7.434	14.922
	1.489	−2.227	−5.933	6.510
	11.309	4.201	−1.369	12.141
	−20.435	13.096	5.839	24.964
	3.004	30.207	−11.124	32.331
Station 1– Station 3	−2.704	5.530	−6.975	9.303
	2.834	−14.163	−1.045	14.481
	−8.297	3.284	−0.450	8.935
	3.356	−0.948	−7.532	8.300
	−12.417	4.447	−4.027	13.790
	−4.803	36.310	−2.077	36.685

**Table 29:** Spatial Distance Calculation Errors of Coordinate Points Solved from Different Measurement Station Pairs.

No.	Point Pair	Reference Distance (mm)	Measured Distance (mm)	Distance Difference (mm)	Mean Error (mm)
Station 1– Station 2	a–d	637.142	637.206	0.064	9.230
	b–e	1209.779	1213.063	3.284	
	c–f	1073.710	1049.369	−24.341	
Station 1– Station 3	a–d	637.142	629.122	−8.020	12.436
	b–e	1209.779	1201.792	−7.987	
	c–f	1073.710	1052.408	−21.302	

## Data Availability

The original contributions presented in this study are included in the article/supplementary material. Further inquiries can be directed to the corresponding author.

## Supplementary Materials

The following supporting information can be downloaded at: https://www.mdpi.com/article/10.3390/s26103177/s1.
